# Effect of Photodynamic Therapy on the Virulence Factors of *Staphylococcus aureus*

**DOI:** 10.3389/fmicb.2016.00267

**Published:** 2016-03-07

**Authors:** Maria Bartolomeu, Sónia Rocha, Ângela Cunha, M. G. P. M. S. Neves, Maria A. F. Faustino, Adelaide Almeida

**Affiliations:** ^1^Departamento de Biologia and Centro de Estudos do Ambiente e do Mar, University of AveiroAveiro, Portugal; ^2^Departamento de Química and Unidade de Investigação em Química Orgânica, Produtos Naturais e Agroalimentares, University of AveiroAveiro, Portugal

**Keywords:** *Staphylococcus aureus*, photodynamic inactivation (PDI), virulence factors, coagulase, enterotoxins, antibiotic/methicillin resistance

## Abstract

*Staphylococcus aureus* is a Gram-positive bacterium that is present in the human microbiota. Nevertheless, these bacteria can be pathogenic to the humans. Due to the increasing occurrence of antibiotic-resistant *S. aureus* strains, new approaches to control this pathogen are necessary. The antimicrobial photodynamic inactivation (PDI) process is based in the combined use of light, oxygen, and an intermediary agent (a photosensitizer). These three components interact to generate cytotoxic reactive oxygen species that irreversibly damage vital constituents of the microbial cells and ultimately lead to cell death. Although PDI is being shown to be a promising alternative to the antibiotic approach for the inactivation of pathogenic microorganisms, information on effects of photosensitization on particular virulence factors is strikingly scarce. The objective of this work was to evaluate the effect of PDI on virulence factors of *S. aureus* and to assess the potential development of resistance of this bacterium as well as the recovery of the expression of the virulence factors after successive PDI cycles. For this, the photosensitizer 5,10,15,20-tetrakis(1-methylpyridinium-4-yl)porphyrin tetra-iodide (Tetra-Py^+^-Me) and six strains of *S. aureus* [one reference strain, one strain with one enterotoxin, two strains with three enterotoxins and two methicillin resistant strains (MRSA) – one with five enterotoxins and the other without enterotoxins] were used. The effect of photosensitization on catalase activity, beta hemolysis, lipases, thermonuclease, enterotoxins, coagulase production, and resistance/susceptibility to methicillin was tested. To assess the development of resistance after successive cycles of treatment, three strains of *S. aureus* (ATCC 6538, 2065 MA, and SA 3 MRSA) were used. The surviving colonies of a first cycle of PDI were collected from the solid medium and subjected to further nine consecutive cycles of PDI. The results indicate that the expression of some external virulence factors is affected by PDI and enterotoxin producing strains were more susceptible to PDI than non-toxigenic strains. The surviving bacteria did not develop resistance. PDI, contrarily to traditional antibiotics, inhibited the expression of virulence factors, efficiently inactivating either highly virulent strains and low virulent *S. aureus* strains, inactivating also antibiotic susceptible and resistant strains, without development of photoresistance after at least 10 consecutive cycles of treatment, and so this therapy may become a strong promising alternative to antibiotics to control pathogenic microorganisms.

## Introduction

*Staphylococcus aureus* is a Gram-positive bacterium that resides on the surface of the skin and on mucous membranes of warm-blooded animals (Morikawa et al., 2001; Costa A.R. et al., 2013) as a commensal microorganism, asymptomatically colonizing the host (Bronner et al., 2004). Nevertheless, due to its invasiveness and taking advantage of host immune weaknesses, *S. aureus* is able to cause a wide spectrum of infections affecting different organs (Bronner et al., 2004; Baptista et al., 2015), from infections of superficial lesions to intoxications and life threatening systemic conditions (Bien et al., 2011). This opportunistic bacterium is a major human pathogen not only associated with community-acquired bacteremia but also nosocomial bacteremia (Morikawa et al., 2001; Cheung et al., 2004; Bien et al., 2011), being readily able to acquire antibiotic resistance (Morikawa et al., 2001). Its ability to survive under stressful conditions, such as those imposed by host immune system, is due to the activation of stress response mechanisms (Morikawa et al., 2001; Bronner et al., 2004; Cheung et al., 2004). These mechanisms involve the action of an interactive regulatory network that includes the accessory gene regulator (*agr*) and staphylococcal accessory element (*sae*) (Bronner et al., 2004; Novick and Geisinger, 2008; Costa A.R. et al., 2013). These two components of the regulatory system regulate the expression of several exoproteins and cell wall-associated proteins related to virulence (Costa A.R. et al., 2013). The regulatory network also includes the staphylococcal accessory regulator A (*sarA*) and its homologs that regulate the expression of some virulence factors; the sigma factors (σ), as the primary sigma factor, σ^A^, that may function in living process through the housekeeping genes expression and the alternative sigma factor σ^B^, which may participate on the bacterial response to stress conditions by regulating the expression of several genes involved on stress response (Morikawa et al., 2001; Cheung et al., 2004; Costa A.R. et al., 2013).

The *S. aureus* pathogenicity involves a wide array of cell wall and extracellular components orderly expressed during the different stages of infection: colonization, avoidance, or invasion of the host immune defense, growth, and cellular division culminating in bacterial dissemination, causing toxic effects to the host (Cheung et al., 2004; Bien et al., 2011; Costa A.R. et al., 2013; Ebrahimi et al., 2014). Some of the cell wall components are responsible for the recognition of adhesive matrix molecules, such as the clumping factor proteins (Clf) that mediates the adherence to fibrinogen (Costa A.R. et al., 2013) and the carotenoid pigment staphyloxanthin that acts as virulence factor once it is able to perform an antioxidant action against oxidant-based reactions (Clauditz et al., 2006; Liu and Nizet, 2009; Costa A.R. et al., 2013). The extracellular components include the superantigen molecules such as the staphylococcal enterotoxins (SE), a family of a single chain proteins with small molecular-weight (24–30 kDa; Johnson et al., 1991; Baptista et al., 2015); the cytolytic β-hemolysin, the clotting factor coagulase, besides more exoenzymes as lipases and nucleases, in which their main function is to disrupt the host cells/tissue and the inactivation of host immune mechanisms of defense (Costa A.R. et al., 2013).

Additionally to the virulence factors already described, *S. aureus* has a notorious capacity to acquire antibiotic resistance (Guillemot, 1999; Morikawa et al., 2001; Ito et al., 2003; Chambers and DeLeo, 2009; Costa A.R. et al., 2013; Theuretzbacher, 2013), by a bacterial gene mutation and horizontal transfer of resistance genes from external sources (Ito et al., 2003; Chambers and DeLeo, 2009). The resistance to penicillin emerged in the mid-1940s, only a few years after the introduction of this antibiotic in the clinical practice (Chambers and DeLeo, 2009; Costa A.R. et al., 2013). Later, in 1959, the semi-synthetic antibiotic methicillin was introduced for the treatment of infections caused by penicillin-resistant *S. aureus* (Enright et al., 2002; Costa A.R. et al., 2013). Yet, in 1961 the first cases of methicillin-resistant *S. aureus* (MRSA) isolates (Chambers and DeLeo, 2009; Costa A.R. et al., 2013) were reported and currently, only few compounds are still effective in the treatment of MRSA infections (Chambers and DeLeo, 2009; Theuretzbacher, 2011).

With the knowledge that the development of new classic antibiotics is not likely to solve the resistance drug problem for too long (Chambers and DeLeo, 2009), non-traditional antimicrobial approaches to treat MRSA infections will be needed. Ideally, the new antimicrobial methods should be non-invasive and non-toxic to the hosts, but efficient and with fast action, avoiding the development of resistance (Calin and Parasca, 2009; Kossakowska et al., 2013; Alves et al., 2014; Almeida et al., 2015). In this context, the photodynamic inactivation (PDI) arises as a photo chemotherapeutic approach with forthcoming applications as antimicrobial therapy (Almeida et al., 2009, 2015; Carvalho et al., 2009; Costa et al., 2012; Alves et al., 2013, 2014, 2015b; Melo et al., 2013). This technology has already proved to be effective against Gram-positive and Gram-negative bacteria, viruses, fungi, and parasites (Almeida et al., 2011, 2015; Costa et al., 2012). The photodynamic effect is based on the use of visible light and an agent (photosensitizer, PS) capable to absorb energy from light and transfer it to molecular oxygen, originating highly cytotoxic species, namely reactive oxygen species (ROS) as singlet oxygen (^1^O_2_), hydrogen peroxide (H_2_O_2_), peroxide anion radical 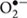, and hydroxyl radical (OH^•^; Alves et al., 2008, 2013, 2014; Calin and Parasca, 2009; Melo et al., 2013). These reactive cytotoxic species can cause irreversible damages to molecular cell constituents or even its destruction (Alves et al., 2014). Initially, the PS adheres to the microbial external structures and later, during the irradiation process, the PDI is initiated and cellular components, such as proteins and lipids will be exposed to oxidizing reactions which will alter their structure and, subsequently, affect the biological function in which they are involved (Alves et al., 2014).

As PDI acts *via* ROS, a high number of microbial targets are simultaneously affected, thus preventing the development of resistance (Costa et al., 2008; Tavares et al., 2010), and allowing the inactivation of a broad-spectrum of microorganisms, independently of their resistance profiles to classic antimicrobials (Tavares et al., 2010; Arrojado et al., 2011; Costa et al., 2011; Almeida et al., 2014). In addition, PDI affects the expression of virulence factors, also causing their degradation (Kömerik et al., 2000; Tubby et al., 2009; Kossakowska et al., 2013). The effects of PDI on virulence factors is of extreme importance as they may be present during the infection process, when the microorganism is present, but they can also be present when the microorganism is not present already, such as in the case of intoxications, causing severe damage to the host.

Clinical trials using light based therapies to evaluate the potential use of photodynamic therapy in the clinical field have been conducted along the last years, with positive results, including studies against viral infections, such as the ones caused by human papilloma virus, with systemic and topical applications (Bujia et al., 1993; Shikowitz et al., 1998) and to treat herpes simplex lesions, with topical applications (Moore et al., 1972). Non-viral infections by *Propionibacterium acnes*, a natural porphyrin producer, were already efficiently inactivated (Kawada et al., 2002; Elman et al., 2003), among others, as acne vulgaris (Ormond and Freeman, 2013). Nowadays the miniaturization of the light devices such as low-power lasers, light emitting diodes, or conventional lamps able to activate the antimicrobial molecules place this technology closer to be use in clinical application. Superficial skin infections like wounds and burns can be easily treated (Ebrahimi et al., 2014). Using fiber-optic technology, most regions of the anatomy are also accessible. *Loci* of infection could be managed endoscopically, allowing local application both of the PS agent and light (Dai et al., 2010; Kharkwal et al., 2011). Even for deep-seated infections a transcutaneous needle could deliver both drug and light via fibers.

Some *in vitro* studies showed that the biological activity of lipopolysaccharides from *Escherichia coli* and proteases from *Pseudomonas aeruginosa* were successfully reduced by toluidine blue (TBO)-mediated PDI (Tubby et al., 2009). Additionally, light-activated methylene blue (MB) showed to inhibit the expression of staphylococcal V8 protease, alpha-hemolysin and sphingomyelinase (Tubby et al., 2009). However, the information about the effects of PDI on virulence factor is still scarce. The objectives of this work were the evaluation of the effect of PDI on some virulence factors of *S. aureus* – catalase activity, beta hemolysis, lipases, thermonuclease, enterotoxins, coagulase – and the assessment of development of resistance to PDI treatment. For that, a cationic porphyrin 5,10,15,20-tetrakis(1-methylpyridinium-4-yl)porphyrin tetra-iodide (Tetra-Py^+^-Me) was used as PS against on six *S. aureus* strains – ATCC 6538, 2153 MA, 2065 MA, 2095 M1A1, DSM 25693 MRSA, and SA 3 MRSA.

## Materials and Methods

### Experimental Design

An experimental procedure was established in order to study the effects of PDI on some virulence factors expression/activity of *S. aureus* strains and to test the potential development of resistance to PDI by *S. aureus* strains after successive photodynamic cycles of treatment, testing also the recovery of the expression/activity of the virulence factors after the successive photodynamic cycles of treatment. Six different strains of *S. aureus*, including methicillin resistant and susceptible strains, as well as, enterotoxin and non-enterotoxin producing strains were tested. The effect of PDI on the expression/activity of virulence factors was tested in all strains. The potential development of resistance to PDI and the recovery of the expression/activity of the virulence factors after the successive cycles of treatment were tested only in three of the strains. For each of these three strains, a total of ten cycles of treatments were performed.

### Characterization of Bacterial Strains and Culture Conditions

Six strains of *S. aureus* were used in this study: ATCC 6538, a non-enterotoxic strain; 2153 MA, the only strain used that does not ferment mannitol (Baptista et al., 2015), producing SE A; 2065 MA, with SE A, G, I, and *S. aureus* 2095 M1A1 with SE C, G and I – the three strains isolated from food products and characterized in the Centre of Biotechnology and Fine Chemistry of the Faculty of Biotechnology of the Catholic University, Porto, Portugal; *S. aureus* DSM 25693, a methicillin-resistant (MRSA) strain, positive for SE A, C, H, G, and I; and a staphylococcal strain isolated from a biological sample from the lower respiratory tract of an hospitalized individual, a non-enterotoxic MRSA strain (SA 3 MRSA; Gonçalves et al., 2014).

All the strains were grown in Brain-Heart Infusion (BHI, Liofilchem, Italy) at 37°C for 18 h at 170 rpm, in order to reach the stationary phase, corresponding approximately to a concentration of 10^8^–10^9^ colony forming units per mL (CFU mL^-1^). Before each PDI assay, a colony of *S. aureus* was transferred to 30 mL of BHI and incubated as previously described. Subsequently, an aliquot was transferred to fresh medium, and grown in the same conditions. This procedure was repeated twice.

### Photosensitizer

The PS 5,10,15,20-tetrakis(1-methylpyridinium-4-yl)porphyrin tetra-iodide (Tetra-Py^+^-Me) used in this study was prepared according to the literature (Carvalho et al., 2010). Their ^1^H NMR and UV–vis spectra were consistent with the literature data. Their purity was confirmed by thin layer chromatography and ^1^H NMR. ^1^H NMR (DMSO-d_6_): -3.12 (s, 2H, *N*H), 4.73 (s, 12H, CH_3_), 9.00 (d, *J* = 6.5 Hz, 8H, Py-o-H), 9.22 (s, 8H, β-H), 9.49 (d, *J* = 6.5 Hz, 8H, Py-m-H). UV–vis (DMSO) λ_max_ (log ε): 425 (5.43), 516 (4.29), 549 (3.77), 588 (3.84), 642 (3.30) nm. The stock solutions (500 μM) of this porphyrin were prepared using the polar aprotic solvent dimethyl sulfoxide (DMSO).

### Antimicrobial Photodynamic Therapy (PDI) Treatments

Bacterial cultures in stationary phase were 10-fold diluted in phosphate buffered saline (PBS) and this bacterial suspension was distributed in sterilized glass beakers. The appropriate quantity of the PS Tetra-Py^+^-Me was added to achieve a final concentration of 5.0 μM. The total volume of final solution was 10 mL per beaker. During the experiments, light and dark controls were also performed: in the light control the beaker without Tetra-Py^+^-Me was exposed to light; in the dark control the beaker containing 5.0 μM Tetra-Py^+^-Me was protected from light with aluminum foil during the experiment. During the pre-irradiation period, the samples were incubated for 10 min with stirring, at room temperature, in order to promote the binding of the porphyrin to *S. aureus* cells. The samples were exposed to an artificial white light (PAR radiation, 13 OSRAM 21 lamps of 18 W each, 380–700 nm) with an irradiance of 40 W m^-2^ for 60 min, under stirring. During the experiment, aliquots of treated and control samples were collected at times 0, 5, 10, 15, 30, and 60 min.

### Enumeration of Viable Cells

From each treated and control samples 10-fold serial dilutions were prepared in sterile PBS (10^-1^ to 10^-6^). Aliquots of 100 μL were pour-plated, in duplicate, in Plate Count Agar medium (PCA, Liofilchem, Italy). The plates were incubated at 37°C for 48 h and the number of colonies was counted. Three independent assays were performed.

### PDI Resistance Assays

In order to verify the development of resistance to PDI treatment with Tetra-Py^+^-Me, ten cycles of PDI were performed. After each cycle of a total irradiation time of 60 min, a new set of bacterial cultures were prepared from an isolated colony, surviving to the previous cycle of PDI (at 37°C, 18 h, 170 rpm). The PDI treatment was repeated under similar conditions. Three independent assays were performed.

### Virulence Factors, Mannitol Fermentation, and Methicillin Susceptibility

To assess if PDI treatments affected the virulence factors of *S. aureus*, treated and controls samples were tested for the presence or activity of virulence factors, according to literature (Baptista et al., 2015). One typical colony of *S. aureus* was selected from each strain. The β-hemolysin activity was detected by streaking Blood Agar Plates (Sheep Blood 7%; BAP, Liofilchem, Italy) and observing the development of a clear/yellow zone surrounding *S. aureus* colonies. Lipase and lecithinase activities were assessed by streaking Baird Park Agar (BPA, Liofilchem, Italy) in which *S. aureus* colonies appear in black, with an opaque precipitation zone (lipase activity) and a clear zone surrounding it (lecithinase activity). The mannitol fermentation was evaluated using Mannitol Salt Agar (MSA, Liofilchem, Italy), being the positive results detected by a change of color of the medium from pink to yellow. Catalase activity was assessed using Catalase/Oxy Test (Liofilchem, Italy), interpreting the formation of gas bubbles as a positive result. The activity of bound coagulase (clumping factor) was determined using Pastorex Staph Plus (Bio-Rad, USA) and the activity of free coagulase was detected using BBL Coagulase Plasma Rabbit (BD, USA). For this, 1.0 mL of supernatant of treated and non-treated samples was collected by centrifugation at 13,000 × *g* for 10 min, at 4°C, and 0.5 mL of BBL Coagulase Plasma reagent was added and the mixture was incubated at 37°C for 24 h. The results were considered positive when the agglutination occurred. The rate of clot formation was evaluated according to the manufacturer’s instruction. Thermonuclease activity was determined by D.N.A. Toluidine Blue Agar (Bio-Rad, USA) and positive results were detected as a change of color of the halos from blue to pink. The presence of SE was determined using SET-RPLA Kit Toxin Detection Kit (Thermo Scientific, UK), a kit based on reversed passive latex agglutination technique, according to the manufacturer’s instruction. Treated and non-treated samples were centrifuged at 900 × *g* for 20 min at 4°C. The presence of enterotoxins H, G, and I was not tested since SET-RPLA Kit Toxin Detection Kit only covers SE A, B, C, and D, which are the most common enterotoxins of *S. aureus* (Baptista et al., 2015). The susceptibility to methicillin was determined using the cefoxitin disk screen test, accordingly to the Clinical and Laboratory Standards Institute [CLSI] (2013). Cultures with halos ≥22 mm were considered methicillin susceptible and cultures with halos ≤21 were classified as methicillin resistant (Clinical and Laboratory Standards Institute [CLSI], 2013). Carotenoid pigments (staphyloxanthin) were determined using a protocol adapted from Morikawa et al. (2001). Each strain was cultured in BHI medium at 37°C for 72 h. Twenty milliliters of the culture were harvested by centrifugation (10,000 × *g*, 10 min) and washed with purified water. The cells were suspended in 5.0 mL of methanol and heated in a bath at 55°C for 15 min, until visible pigments have been extracted. Cellular debris was removed by centrifugation at 15,000 × *g* for 10 min. The absorbance at 465 nm of the methanol extracts were measured in a quartz cuvette in a spectrophotometer (Dynamica Halo DB-20, UK).

The β-hemolysis, lipase and lecithinase, catalase, bound coagulase and thermonuclease activities, mannitol fermentation and methicillin susceptibility were tested in the PDI surviving cells, after plating and incubation at 37°C. Methicillin susceptibility was inferred from the diameter of the inhibition zone around cefoxitin disks, and the results represent the average of the inhibition zones from three independent tests. Free coagulase and SE A and C activities were assessed in the supernatant of treated samples and controls after PDI assays.

The effect of PDI on the isolated toxins was also individually assessed. Purified SE A and C (available on SET-RPLA Kit Toxin Detection Kit as SE A and C controls) were subjected to the PDI treatment. The concentration of the PS was the same used before: 5.0 μM; the amount of SE used was 63 μL from the reconstituted control reagents (the kit control solution contains 25 ng of purified enterotoxin reconstituted in 0.5 mL of diluent from the kit as showed in Figure in section “Quantity of Enterotoxins (ng) Per Kit” of the Supplementary Material), and PBS was added to make up a total volume of 2.0 mL of solution, which was irradiated. Light and dark controls were included. At times 0, 5, 10, 15, 30, and 60 min aliquots of 25 μL were collected and the activity of the SE was tested using the SET-RPLA Kit Toxin Detection Kit.

## Results

### Bacterial Inactivation by PDI

**FIGURE 1:**
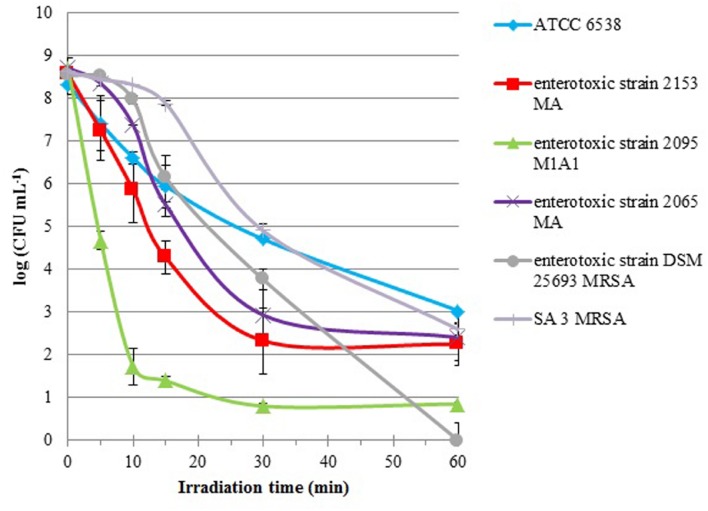
**Survival curves of six *S. aureus* strains (ATCC 6538, enterotoxic strains 2153 MA, 2095 M1A1, 2065 MA, and DSM 25693 MRSA and SA 3 MRSA) incubated with 5.0 μM of Tetra-Py^+^-Me and irradiated with white light (380–700 nm) with an irradiance of 40 W m^-2^ for 60 min.** Lines representing light (LC) and dark (DC) controls viability were omitted, since cell viability in the controls was not affected. Values represent the mean of three independent experiments with two replicates each; error bars indicate the standard deviation.

Cells suspensions of *S. aureus* strains were subjected to 60 min of PDI treatments (5.0 μM of Tetra-Py^+^-Me and an irradiance of 40 W m^-2^), and aliquots were taken before (0 min) and after 5, 10, 15, 30, and 60 min of treatment. All the strains were efficiently inactivated by PDI (**Figure 1**). After 60 min of treatment under the tested conditions reductions higher than 5 log CFU mL^-1^ were observed for all the tested strains. However, in general, the pattern of photoinactivation was different among the *S. aureus* strains as showed by the log10 reduction rates (**Figure 1**).

The enterotoxic strains were more efficiently inactivated than the non-enterotoxic ones. The toxigenic *S. aureus* strain 2095 M1A1 was particularly susceptible to PDI showing a reduction of 7 log within the first 10 min of irradiation. With equivalent irradiation time, the enterotoxic strain 2153 MA showed a reduction of only 2.5 log and all the other strains were inactivated less than 2 log. For DSM 25693 MRSA strain, also an enterotoxic, a linear decrease was observed after 15 min of treatment, reaching complete inactivation after 60 min of treatment (8.5 log). After 60 min of treatment, the inactivation factors for the other strains were 5.3 log for ATCC 6538, 6.0 log for SA 3 MRSA, 6.3 log for 2065 MA, 6.3 log for 2153 MA, and 7.8 log for 2095 M1A1. In the controls, the concentration of viable cells did not vary, indicating that the viability of bacterial cells was neither affected by light alone (light control), nor by the direct toxicity of the PS (dark control).

### Development of Resistance after Repeated PDI Cycles and Recovery of Viability Between Cycles

**FIGURE 2:**
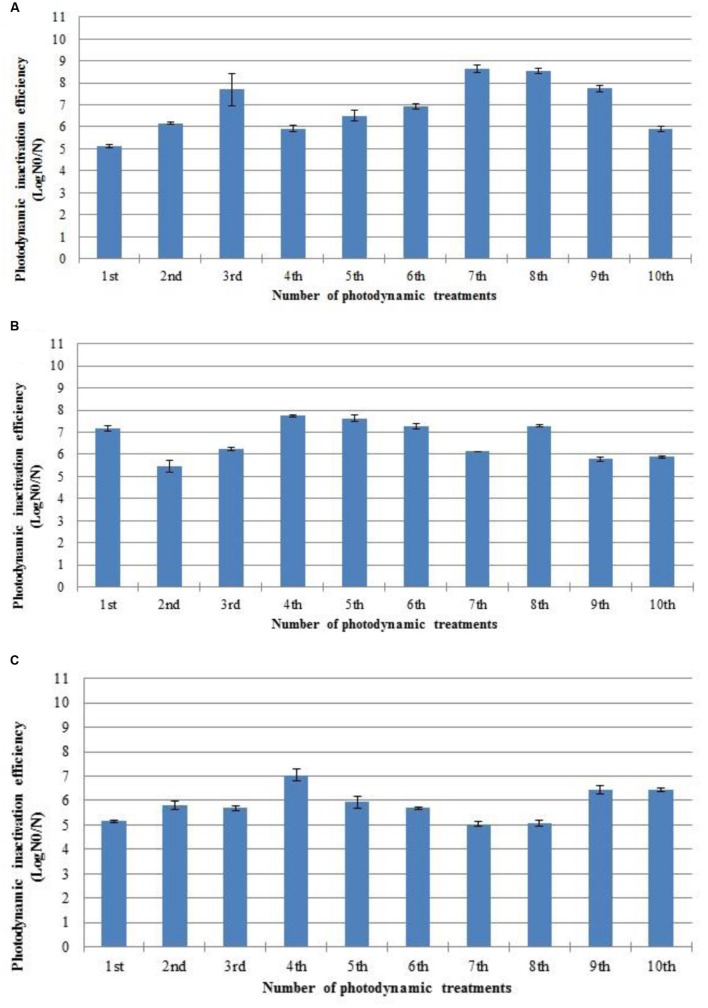
**Photodynamic inactivation efficiency of ten consecutive cycles of *S. aureus* ATCC 6538 **(A)**, 2065 MA **(B)**, and SA 3 MRSA **(C)** by 5.0 μM of Tetra-Py^+^-Me after 60 min of irradiation with white light (40 W m^-2^).** N_0_ represents the plaque counts of bacterial cells before the irradiation; N represents the plaque counts after the cycle treatment; error bars indicate the standard deviation.

Three *S. aureus* strains (ATCC 6538, 2065 MA, and SA 3 MRSA) were subjected to ten consecutive PDI cycles (**Figure 2**). The authors decide to test 10 cycles having into account previous results of the research group (Tavares et al., 2010; Costa et al., 2011). The PDI efficiency of photosensitization with Tetra-Py^+^-Me was not affected during the sequence of 10 PDI cycles.

### Effect of PDI on *S. aureus* Virulence Factors, Methicillin Susceptibility, and Mannitol Fermentation

After each PDI assay, the activity and presence of virulence factors, mannitol fermentation, and methicillin susceptibility were tested (**Table 1**).

**Table 1:** The activity of the virulence factors, mannitol fermentation, and susceptibility to methicillin were tested, after PDI treatments, in presence of 5.0 μM of Tetra-Py^+^-Me and irradiated with white light (380–700 nm) with an irradiance of 40 W m^-2^ for 60 min.

*Staphylococcus aureus* strains	Samples	Surviving cells							Supernatant		
			
		β-Hemolysis	Lipase and lecithinase	Mannitol fermentation	Catalase	Bound Coagulase	Thermonuclease	Susceptibility to methicillin (mm)	Free coagulase	SEs
										
										A	C
ATCC 6538	S	+	+	+	+	+	+	30	–	#	#
	LC	+	+	+	+	+	+	29	4^+^	#	#
	DC	+	+	+	+	+	+	29	4^+^	#	#
2153 MA	S	+	+	–	+	+	+	25	–	–	#
	LC	+	+	–	+	+	+	26	3^+^	+	#
	DC	+	+	–	+	+	+	26	3^+^	+	#
2065 MA	S	+	+	+	+	+	+	28	–	–	#
	LC	+	+	+	+	+	+	28	3^+^	+	#
	DC	+	+	+	+	+	+	28	3^+^	+	#
2095 M1A1	S	+	+	+	+	+	+	27	–	#	–
	LC	+	+	+	+	+	+	26	3^+^	#	+
	DC	+	+	+	+	+	+	27	3^+^	#	+
DSM 25693 MRSA	S	+	+	+	+	+	+	<21	–	–	–
	LC	+	+	+	+	+	+	<21	3^+^	+	+
	DC	+	+	+	+	+	+	<21	3^+^	+	+
SA 3 MRSA	S	+	+	+	+	+	+	<21	–	#	#
	LC	+	+	+	+	+	+	<21	4^+^	#	#
	DC	+	+	+	+	+	+	<21	4^+^	#	#


*These tests were performed in three independent assays for each strain. Cardinal symbol in the “Supernatant, SE” columns means that such tests have not been performed for the respective strains once these strains do not produce the mentioned virulence factors.*

The surviving cells to PDI treatments retained the capacity to express all the virulence factors and to ferment mannitol. However, the activity of the extracellular virulence factors free coagulase and enterotoxins, assessed in the supernatant of treated samples, was affected (see section “Testing the Presence of Free Coagulase” of Supplementary Material and **Table 1**). For the test of free coagulase, the BBL Coagulase Plasma reagent was added to an aliquot of supernatant and the resulting samples was incubated for 24 h. Clot formation was not detected in photosensitized samples. The SE test, performed in the supernatant of photosensitized cells by the SET-RPLA Kit Toxin Detection Kit test, revealed the formation of a tight button, interpreted as absence of SE or presence at a concentration below the detection limit. These two virulence factors persisted in light and dark controls.

### Susceptibility of Staphylococcal Isolated Enterotoxins to PDI

The isolated enterotoxins A and C were directly treated by PDI [see section “Purified SE A (A) and C (B) Subjected to Photodynamic Treatment” of Supplementary Material and **Table 2**]. The positive result corresponds to agglutination, leading to the formation of a lattice structure and negative correspond to the formation of a tight button, which occurs if SE are absent or present in a concentration bellow the detection level (TD0900 SET-RPLA, 2015). Before the treatment, enterotoxins A and C were still detected (formation of a lattice structure). During the PDI, the formation of the lattice structure decreases (as seen in the first well of 15 and 30 min test, see section “Purified SE A (A) and C (B) Subjected to Photodynamic Treatment” of Supplementary Material) and the formation of a tight button (as seen in the first well of 60 min test), begins to occur, indicating the shift to a negative result. After 60 min of irradiation, the inactivation was >68% for both SE [SE A and C; see section “Calculating the Decrease (%) of Enterotoxins After PDI Assays” of Supplementary Material].

**Table 2:** The purified SE A and C were subjected to PDI for 60 min.

Irradiation time		Isolated and purified SEs	
		
		A	C
0	S	+	+
	LC	+	+
	DC	+	+
60	S	–	–
	LC	+	+
	DC	+	+


*The SE activity was assessed before (0 min), during (5, 10, 15, and 30 min) and after 60 min of treatment for treated (S) and non-treated samples (light control, LC; dark control, DC). Three independent assays were performed.*

### Carotenoid Pigments Content

Carotenoid pigments were detected in all the tested strains. The strains SA 3 MRSA with an absorbance at 465 nm of 0.995 ± 0.001 and the 2065 MA with an absorbance of 0.911 ± 0.017, display the highest concentration of pigments, followed by DSM 25693 MRSA (Abs of 0.788 ± 0.021), ATCC 6538 (Abs of 0.701 ± 0.005), 2153 MA (Abs of 0.480 ± 0.002), and 2095 M1A1 (Abs of 0.411 ± 0.013).

## Discussion

According to literature, the susceptibility of *S. aureus* to PDI is strain-dependent and MRSA strains seem to have a lower susceptibility to PDI than methicillin-sensitive counterparts (Grinholc et al., 2008). The lower susceptibility of MRSA strains to PDI has been attributed to slime production by the MRSA strains used (Grinholc et al., 2008).

In this study, all the strains tested were susceptible to PDI, with a survival reduction above 5 log CFU mL^-1^, which according to American Society of Microbiology is higher than the minimum required (reduction > 3 log CFU mL^-1^) for a new approach to be termed as antimicrobial (ASM, 2015). However, as observed in previous studies, a strain-dependent efficiency of inactivation was observed (Grinholc et al., 2008). Nonetheless PDI does not seem to be antibiotic-susceptible dependent, since DSM 25693, an MRSA and enterotoxic strain, was the only strain that was inactivated to the limit of detection of the method, with a survival reduction of 8.5 log. Another MRSA strain, SA 3 MRSA, was not as efficiently inactivated, but the inactivation profile was similar to that of the reference, ATCC 6538 strain. The four enterotoxic strains were more efficiently inactivated that the two non-enterotoxic strains (see **Figure 1**), which could suggest that the enterotoxic strains are more susceptible to PDI. However, as showing the log10 reduction rates differences in PDI inactivation between enterotoxic strains were observed, which means that other cell factors may contribute to these differences in PDI efficiency. Whereby, further studies should be performed exploiting the factors which may contribute to the difference in PDI susceptibility among the bacterial strain.

Carotenoid pigment content is related with the resistance of *S. aureus* to several stress factors, such as external oxidative stress. Several studies have shown that mutant strains that are unable to produce carotenoids are more susceptible to those stress factors (Liu et al., 2005; Clauditz et al., 2006; Cebrián et al., 2007). In an attempt to verify if carotenoid pigments were able to attenuate oxidative damages and consequent cellular inactivation, the pigment content of each strain was measured. Although a direct relation between carotenoids content and PDI efficiency was not observed for all the strains, the least susceptible strains to PDI (ATCC 6538 and SA 3 MRSA) show a high relative concentration of this pigment, with absorbances of 0.701 and 0.995, respectively.

Once the photodynamic action occurs through ROS generation (formed along the irradiation process), the damages (through oxidative process) can affect a variety of cellular components with great importance in the maintenance of bacterial stability, such as molecular components (proteins and lipids) of external structures, and enzymes (Alves et al., 2014; Almeida et al., 2015). The majority of virulence factors of *S. aureus* are proteins or enzymes that are prone to photodynamic oxidative damage. The results of this study show that the phenotypic expression of the of β-hemolysin, lipase, lecithinase, catalase and bound coagulase by surviving cells cultivated on PCA medium for 48 h at 37°C was not affected by PDI. Mannitol uptake and fermentation also persisted. Previous proteomic analysis showed that the oxidative damage caused by PDI treatment affects the expression of functional proteins involved in cell division, metabolic activities, oxidative stress responses, and sugar uptake (Alves et al., 2014, 2015a). However, the mentioned studies were performed in treated cells. Since the detection of virulence factors activity in our study was performed in the surviving cells after a period of recovery, it would be expected that the bacteria, even if injured during PDI treatments, would be able to continue producing those virulence factors and other enzymes.

Antioxidant enzymes such as superoxide dismutase, catalase, and peroxidase can give protection against some ROS but not against singlet oxygen. So, according to the literature porphyrinic compounds (Almeida et al., 2011), namely the tetracationic porphyrin Tetra-Py^+^-Me used in this study, exert their photodynamic action by a type II mechanism and consequently singlet oxygen is the main ROS (Tavares et al., 2011; Costa L. et al., 2013). Moreover, has been shown that singlet oxygen is able to inactivate these enzymes, namely the catalase enzyme (Kim et al., 2001). However, no effect of the PDI process was observed for this enzyme, once that in this study, the catalase activity was only determined after a recovery period of the persistent cells, and not during the PDI treatment – condition in which could be detected some changes in the activity of catalase. Further studies are needed to clarify whether the ROS produced by the Tetra-Py^+^-Me during PDI treatments affect catalase enzyme and even other enzymes when they are outside the cells.

Nevertheless, some of these virulence factors are released to the extracellular compartment, becoming more exposed to the effects of the PDI process suffering a greater damage.

In this study, the presence/activity of two external virulence factors (free coagulase and enterotoxins A and C) in the supernatant of PDI treatment samples was assessed. The results show that both virulence factors are affected by PDI. This represents an advantage relatively to traditional antibiotics, which act only on bacterial cell and not on extracellular virulence factors. These results are in accordance with those observed in the previous studies by Kömerik et al. (2000) and Tubby et al. (2009).

The response of one of the most important *S. aureus* virulence factors, the SE which are the staphylococcal food poisoning causative agents, was also assessed after PDI treatment in order to confirm the effect of PDI on extracellular virulence factors. Two isolated enterotoxins, SE A and C, were treated by PDI. The porphyrin Tetra-Py^+^-Me at a concentration of 5.0 μM reduced at least in 68% the amount of active SE A and C. This discovery is of great importance since it is known that this family of proteins covers very stable, resistant to heat and to degrading enzymes molecules (Schelin et al., 2011). These results demonstrate that PDI is not only effective in the inactivation of microorganisms but also in the degradation of released external virulence factors.

One of the main advantages of PDI is that because of the nature of the photoinactivation process, development of resistance is very unlikely (Tavares et al., 2010; Costa et al., 2011). The results obtained in this study do not show evidence of resistance development in the three strains subjected to ten PDI cycles, which corroborates literature conclusions. Considerable reduction in the efficiency of photosensitization of *S. aureus* strains ATCC 6538, enterotoxic 2065 MA and SA 3 MRSA after ten consecutive photosensitization sessions of 60 min with 5.0 μM of Tetra-Py^+^-Me was not observed. As in this study, the viable bacterial colonies have been aseptically removed from the plate and suspended in PBS after each PDI cycle, the cellular density obtained after the colony resuspension could be different. To avoid differences in the PDI efficiency due to different bacterial densities, this parameter was controlled in all the experiments by measuring the optical density of the bacteria suspension before each assay.

## Conclusion

Overall, it can be concluded that (1) although the efficiency of PDI to inactivate *S. aureus* is strain-dependent, all the strains can be effectively inactivated, namely the enterotoxic strains; (2) PDI process is not only effective in the inactivation of microorganisms but also in the degradation of their external virulence factors after their release to the exterior; and (3) *S. aureus* strains do not develop resistance to PDI treatment.

The research conducted so far, allowed the development of very promising and successful *in vitro* photoinactivation protocols for enterotoxigenic and non-enterotoxigenic strains of *S. aureus*. However, their evaluation *in vivo*, along with validation of their feasibility according to different sample settings and different enterotoxigenic and non-enterotoxigenic strains of *S. aureus*, as well as on biofilms developed by these bacteria, are now required. Bacterial biofilms are not only more difficult to inactivate than planktonic bacteria, but bacteria in a biofilm-mode are also much more prone to become resistant to antimicrobial agents or strategies (Beirão et al., 2014). In fact, some mechanisms, such as horizontal gene transfer and quorum sensing, occur only in a biofilm-state (Madsen et al., 2012; Solano et al., 2014).

## Author Contributions

MB did the experimental work and drafted the manuscript. SR participated in the experimental work. AA has been involved in the coordination, conception, design of the study and helped to draft the manuscript. ÂC, MF, and MN participated in the design of the study, acquisition and interpretation of data, and also helped.

## Conflict of Interest Statement

The authors declare that the research was conducted in the absence of any commercial or financial relationships that could be construed as a potential conflict of interest.
