# Supplementary material for: Effect of Photodynamic Therapy on the Virulence Factors of Staphylococcus aureus
Source: Front Microbiol. 2016 Mar 7;7:267. doi: 10.3389/fmicb.2016.00267 (PMC4780358; doi:10.3389/fmicb.2016.00267)

Supplementary Material

Effect of photodynamic therapy on the virulence factors of Staphylococcus aureus

Maria Bartolomeu, Sónia Rocha, Ângela Cunha, M. Graça P. M. S. Neves, M. Amparo F. Faustino, Adelaide Almeida*

*** Correspondence:** aalmeidaa@ua.pt

# 1. Supplementary Data

**1.1 Quantity of enterotoxins (ng) per kit**

**
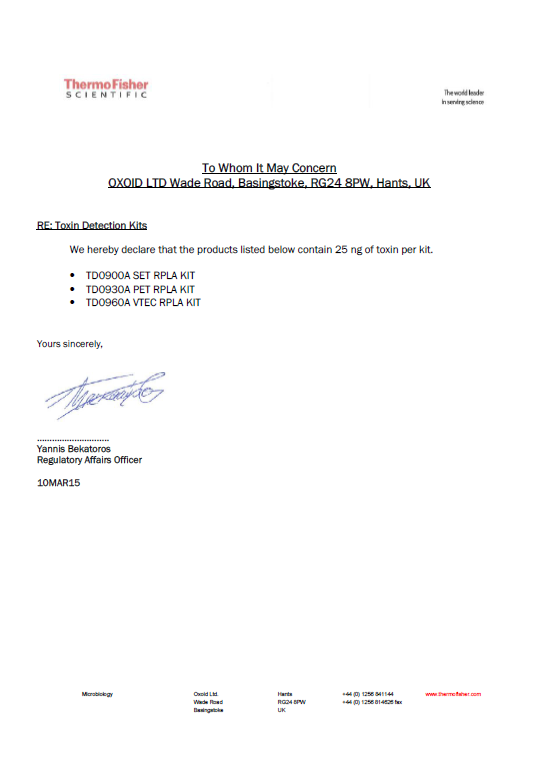
**

**1.2 Calculating the decrease (%) of enterotoxins after PDI assays**

Amount of enterotoxin in control of the SET-RPLA Kit Toxin Detection Kit: 25 ng.

This amount is reconstituted in 0.5 mL of diluent. Hence, the concentration of enterotoxins in control solutions is 50 ng.mL^-1^.

63 µL of control solutions were used to integrate irradiated samples and the light (LC) and dark (DC) controls. In each 63 µL there are 3.15 ng of enterotoxin.

The total volume of irradiated samples was 2.0 mL (63 µL of enterotoxin control solution + 20 µL of **Tetra-Py^+^-Me** of stock solution to achieve a concentration of 5.0 µM + 1917 µL of PBS) with a concentration of enterotoxin of 1.58 ng.mL^-1^.

Were collected aliquots of 25 µL from the total volume of irradiated samples to test the presence of enterotoxins. In the first well of the top three rows (SEA and LC and DC controls before treatment) in the Figure 4, the enterotoxins are present at a concentration of 1.58 ng.mL^-1^. In the first well of the 8^th^ row (sample after 60 min of treatment) the result is negative (formation of a tight button) for the presence of enterotoxin. It means that the enterotoxin is absence or present at concentrations bellow the method detection limit.

In the Instruction leaflet of the kit it is written (in section Limitations of the Test) that “The sensitivity of this test in detecting the enterotoxins has been reported to be 0.5ng/mL in the test extract”. So, assuming that in the first well of the 8^th^ row the enterotoxins are present at a concentration ≤ 0.5 ng.mL^-1^, there was a decrease of enterotoxin concentration of at least three times along the 60 min of treatment.

In percentage, it means a decrease of at least 68%.

**1.3** **Testing the presence of free coagulase.**

After each PDI assay was assessed if free coagulase (an extracellular virulence factor) was present in the supernatant of samples involved in the assays. The clot formation indicates a positive result; a negative result occurs when there is no clot formation. [A60’, treated sample (5.0 µM of Tetra-Py^+^-Me and light), after 60 min of treatment; CC60’, irradiated but non-treated (only light); CE60’, non-irradiated sample (only 5.0 µM of Tetra-Py^+^-Me].

**
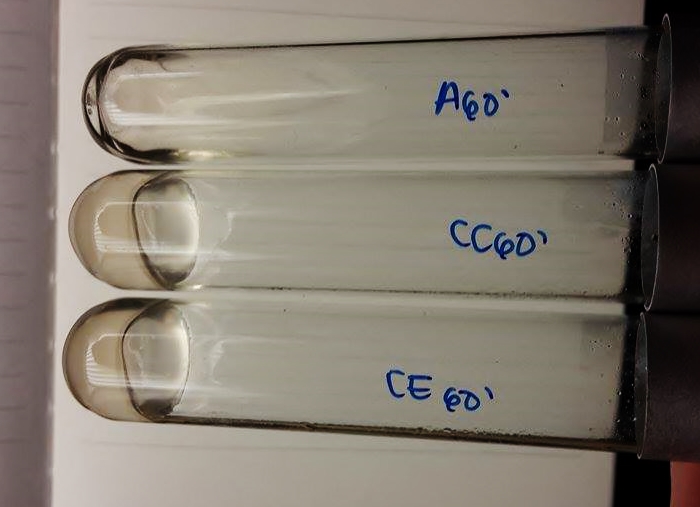
**

**1.4** **Purified SE A (A) and C (B) subjected to photodynamic treatment.**

The purified SE A (A) and C (B) were subjected to photodynamic treatment, in presence of 5.0 µM of Tetra-Py^+^-Me and irradiated with white light (380–700 nm) with an irradiance of 40 W m^−2^ for 60 min. Rows represent a different sample; columns represent a different dilution (doubling dilutions). In the first three rows was tested the presence of SE before the treatment (SE A 0’ and C 0’, red arrows); in the following four rows were tested the presence of SE after 5, 10, 15 and 30 min of treatment; in the last three rows were tested the presence of SE after the treatment (a total of 60 min) (SE A 60’ and C 60’, blue arrows). Three independent assays were performed for each SE.


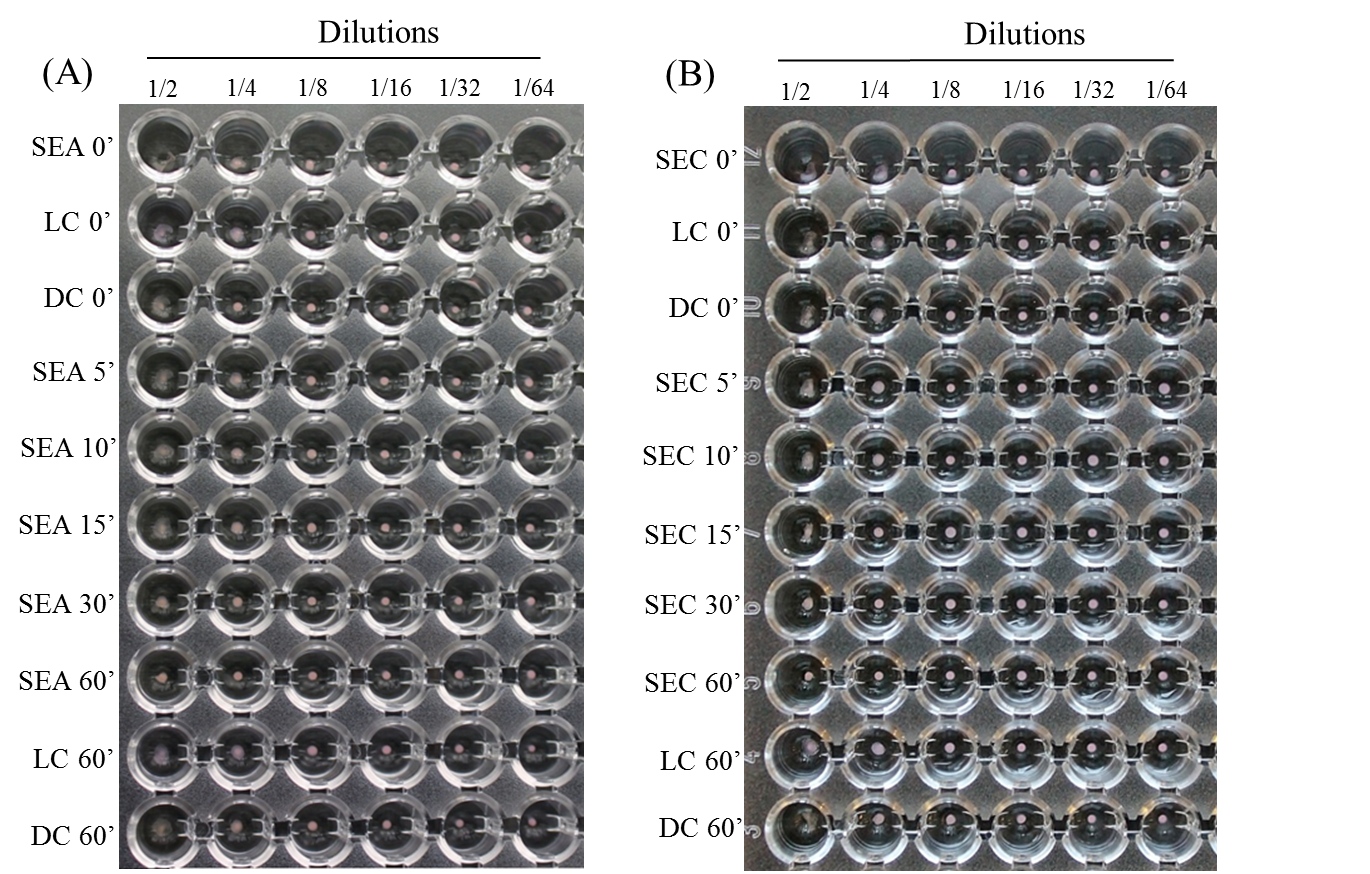

Supplement: Supplementary file 1 [file Data_Sheet_1.DOCX]
